# Influence of protoplast fusion between two *Trichoderma* spp. on extracellular enzymes production and antagonistic activity

**DOI:** 10.1080/13102818.2014.978206

**Published:** 2014-11-14

**Authors:** Mohamed M. Hassan

**Affiliations:** ^a^ Scientific Research Center, Biotechnology and Genetic Engineering Unit, Taif University, Taif, KSA; ^b^ Genetics Department, Faculty of Agriculture, Minufiya University, Shebin El-Kom, Egypt

**Keywords:** *Trichoderma*, protoplast fusion, β-glucanase, chitinase, protease, antagonistic activity, grapevine pathogens

## Abstract

Biological control plays a crucial role in grapevine pathogens disease management. The cell-wall degrading enzymes chitinase, cellulase and β-glucanase have been suggested to be essential for the mycoparasitism activity of *Trichoderma* species against grapevine fungal pathogens. In order to develop a useful strain as a single source of these vital enzymes, it was intended to incorporate the characteristics of two parental fungicides tolerant mutants of *Trichoderma* belonging to the high chitinase producing species *T. harzianum* and the high cellulase producing species *T. viride*, by fusing their protoplasts. The phylogeny of the parental strains was carried out using a sequence of the 5.8S-ITS region. The BLAST of the obtained sequence identified these isolates as *T. harzianum* and *T. viride*. Protoplasts were isolated using lysing enzymes and were fused using polyethylene glycol. The fused protoplasts have been regenerated on protoplast regeneration minimal medium supplemented with two selective fungicides. Among the 40 fast growing fusants, 17 fusants were selected based on their enhanced growth on selective media for further studies. The fusant strains were growing 60%–70% faster than the parents up to third generation. All the 17 selected fusants exhibited morphological variations. Some fusant strains displayed threefold increased chitinase enzyme activity and twofold increase in β-glucanase enzyme activity compared to the parent strains. Most fusants showed powerful antagonistic activity against *Macrophomin aphaseolina*, *Pythium ultimum* and *Sclerotium rolfsii* pathogens. Fusant number 15 showed the highest inhibition percentage (92.8%) against *M. phaseolina* and *P. ultimum,* while fusant number 9 showed the highest inhibition percentage (98.2%) against the growth of *S. rolfsii.* A hyphal intertwining and degradation phenomenon was observed by scanning electron microscope. The *Trichoderma* antagonistic effect against pathogenic fungal mycelia was due to the mycoparasitism effect of the extracellular enzymes.

## Introduction

Genus *Trichoderma* is one of the most important filamentous fungal biocontrol agents in agriculture for the management of plant diseases caused by a wide spectrum of fungal pathogens.[1,2] Many species under this genus such as *T. harzianum, T. viride* and *T. hamatum* have been used against diseases in a wide variety of economically important crops such as grapevine.[3,4] *Trichoderma*, being a saprophyte adapted to thrive in diverse situations, produces a wide array of enzymes such as chitinase and cellulolytic enzymes. Production of extracellular hydrolytic enzymes is one of the biocontrol mechanisms exerted by *Trichoderma* towards fungal pathogens.

Among *Trichoderma* species, *T. harzianum, T. viride* and *T. reesei* are known as producers of these extracellular hydrolytic enzymes. *T. harzianum* is a known producer of the extracellular hydrolytic enzyme, chitinase, which is one of the biocontrol mechanisms exerted by *Trichoderma* towards fungal pathogens, besides antibiosis and competition, which degrades the chitin polymers of the fungal cell wall.[5] Chitinase was used for biological control of fungal diseases.[2,6,7] In industry, chitinase enzymes are extensively used for the degradation and other processes of cellulose materials particularly in textile and paper. It was also used for wastewater treatment.[7]

*T. viride* is a known producer of cellulolytic enzymes.[8,9] It can secrete an enzyme system capable of degrading crystalline cellulose, which consists of several cellobiohydrolases, endoglucanases and β-glucosidases. These enzymes are employed for the conversion of wood, cellulosic agricultural by products to fermentable substances and in the conversion of lignocellulosic materials into biofuels (like ethanol) and single cell proteins. By selecting strains that produce a particular kind of enzyme and culturing these in suspension, industrial quantities of enzyme can be produced.[9,10] However, further improvement of these strains and enhancement of their antagonistic activity can be done using the modern techniques that offer a valuable means for more direct approaches.[11,12]

Protoplast fusion is one of the important approaches in the fungal strain improvement technique.[13,14] Fungal protoplasts are important tools in physiological and genetic research, as well as genetic manipulation which can be successfully achieved through fusion of protoplasts in filamentous fungi that lack the capacity for sexual reproduction.[5,15] Protoplast fusion facilitates the transfer of mitochondrial genomes between taxonomically related species.[16] It can be viewed as one of the recombinant DNA technology that provides the tools for increasing the gene dosage and gene expression from strong promoters, deletion of unwanted genes from the fungal genome, manipulation of metabolic pathways and developing fungal strains for the production of heterologous proteins.

Isolation, fusion and regeneration of protoplasts have been achieved in the genus *Trichoderma* mainly to enhance its cellulolytic activity [14,17,18] and chitinase production.[8] However, limited attempts were made to improve the strain of *Trichoderma* and to enhance enzyme production.[7,18] Accordingly, there is a strain improvement potential in *Trichoderma* for enhancing extra-cellular enzymes and antagonistic activity especially against some grapevine pathogens.

Several molecular techniques used to characterize fungi species were reported, including restriction fragment length polymorphism, random amplified polymorphic DNA and isozymes analysis.[7,19] Furthermore, sequence analysis of the nuclear ribosomal internal transcribed spacer of rDNA (ITS-rDNA region) is one of the famous methods among these molecular characterization techniques. The rRNA genes are universally conserved, while the ITS region and intergenic spacer are highly variable.

With this background, the objective of our study was: (1) molecular identification of two parental isolates of *Trichoderma* that are initially selected for their fungicide tolerance. (2) Fuse their protoplast with the objective of investigating the possible enhancement of the extracellular cellulase, chitinase and protease production in the fusant progenies. (3) Evaluate the fusants antagonistic activity against some soil pathogens.

## Materials and methods

### Materials

This study was done during the period from January 2012 to May 2013 in Scientific Research Center, Biotechnology and Genetic Engineering Unit, Taif University, KSA. The two fungicides benomyl and carbendazim were obtained from Oxford Chemicals Limited (OCL) (UK). Protoplast lysing enzymes L1412 from *Trichoderma harzianum* were obtained from Sigma Co. (Sigma-Aldrich Chemie GmbH). All other chemicals were of the commercially available highest grade.

### Strains

Two mutant strains of *Trichoderma* spp. that showed a tolerance for the two fungicides benomyl and carbendazim, respectively, were obtained as a kind gift from Scientific Research Center, Biotechnology and Genetic Engineering Unit, Taif University, KSA. Further molecular identification of these strains at species level was done. The species were positively identified as *T. harzianum* and *T. viride.*

### Media

Potato dextrose agar medium (PDA) was used as growth and maintenance medium for fungal cultures. Basal salt media containing enzyme substrate were used for the production of the studied enzymes. The basal medium [20] containing 100 μg ampicillin/mL was used as growth medium for protoplast isolation. Protoplast regeneration minimal medium (PRMM) was used for protoplast regeneration as described previously.[21]

### Molecular characterization of *Trichoderma* isolates

#### Genomic DNA isolation

Fungal mycelia of the selected fungus strains were inoculated onto PDA broth for five days. Genomic DNA for each *Trichoderma* strain was extracted using DNeasy Plant Mini Kit (QIAGEN) according to the manufacturer's instructions.

### PCR amplification of 5.8S-ITS region

ITS1 and ITS2 regions together with the 5.8S gene in rRNA from both species were amplified using the primer pair of ITS-1 (5′-TCC GTA GGT GAA CCT GCG G-3′) and ITS-4 (5′-TCC TCC GCT TAT TGA TAT GC-3′) as designed by Hermosa et al. [22] with some modifications. PCR amplification was conducted in 25 μL reaction mixtures containing 1X PCR buffer (DreamTaq™), 2 mmol/L MgCl_2_, 0.08 μmol/L of each primer, 160 μmol/L of each deoxynucleotide triphosphate, 1.25 U of Taq DNA polymerase (DreamTaq™ DNA Polymerase) and about 10 ng of genomic DNA. PCR amplification was carried out in a TPersonal thermocycler (Bio-rad) programmed as follows: an initial denaturation for 5 minutes at 94 °C, followed by 35 cycles of denaturation at 94 °C for 1 minute, annealing of primers at 56 °C for 45 seconds, and extension at 72 °C for 30 seconds, and the amplification was completed with one cycle of final extension at 72 °C for 5 minutes.

### Sequence analysis of 5.8S-ITS region

The nucleotide sequences of 5.8S-ITS region were determined using the sequencer (Gene analyzer 3121). The deduced sequence was aligned using Molecular Evolutionary Genetics Analysis (MEGA) version 5.10. The forward and reverse sequences were checked and edited manually when needed. Then, a consensus sequence was generated from each alignment made. The sequencing data were compared against the GenBank database (http://www.ncbi.nlm.nih.gov/BLAST/), where a nucleotide blast program was chosen to identify the homology between the PCR fragments and the sequences in the GenBank database. In addition, the 5.8S-ITS sequences were compared to a *Trich*OKEY 2 program.

### Protoplast isolation

Protoplasts were prepared from actively growing mycelium of the two fungicide tolerant isolates of *Trichoderma* using enzyme lysis as previously described.[6] The protoplasts were counted on a haemocytometer and diluted to 1 × 10^5^ mL^−1^.[15]

### Protoplast fusion

Protoplasts were fused using a procedure similar to that described by Pe’er and Chet [21] with about equal number of protoplast from two different mutants of two different strains.

### Protoplasts regeneration

The fusion mixture was serially diluted with the osmotic stabilizer and plated on PRMM selective media (media amended with fungicides) and checked for regeneration and fusion of protoplast.

### Morphological characterization

The non-fusion parents and fusants were grown on PDA media and their colony morphology, pigmentation and sporulation were observed. The growth pattern morphology and sporulation were studied by cover slips technique according to Srinivasan et al.[14]

### Enzyme assays

Chitinase (EC 3.2.1.14) and β-1,3-glucanase (EC 3.2.1.39) activity were assayed by the colorimetric method using colloidal chitin and laminarin as substrates, respectively. The level of reducing sugar released was determined by the Dinitrosalicylic acid (DNSA) method, using *N*-acetylglucosamine and glucose as a standard.[11] Reactions were conducted for 30 minutes at 55 °C.[23] Specific activity of chitinase and β-1,3-glucanase was expressed as 1 μmol of reducing sugars released × h^−1^ × mg^−1^ protein. Non-enzymatic controls were also performed using boiled enzymes and were subtracted from the enzymatic values. Protease activity (EC 3.4.21.4) was measured using casein as a substrate. The reaction was carried out according to Gajera et al. [24], blank was treated as zero-time incubation. The amount of released total free amino acids was estimated by Ninhydrin method. Proteolytic activity was corresponded to the amount of enzyme required to cause an increase of μg (free amino acids) × h^−1^ × mg^−1^ protein in culture supernatant. Total phenol content was estimated as method described by Gajera et al. [24] and calculated as μg × ml^−1^ culture supernatant using pyrocatechol as a standard. Concentrations of released fungal extracellular proteins were determined using modified Bradford method.[25]

### Antagonistic test against some grapevine pathogens

Parental strains of *T. harzianum*, *T. viride* and their fusants were subjected to test of biocontrol activity against *Macrophomina phaseolina*, *Pythium ultimum* and *Sclerotium rolfsii* by dual culture technique on PDA medium according to the method described by Fahmi et al.[6] The potential biocontrol agent *Trichoderma* spp. and the pathogens were point inoculated 3 cm apart on PDA medium plates. Parents and fusants of *Trichoderma* were prepared to study interaction of each of the 19 of *Trichoderma* with three isolates of pathogens. The ability of antagonistic activity of *Trichoderma* strains against these pathogens were measured after five days of incubation at 28 °C. The percentage of inhibition (*I*%) on the mycelial growth of pathogens were calculated using this formula: percentage of inhibition (*I*%) = [(*R*1 − *R*2)/*R*1] × 100, where *R*1 = radius of the pathogen away from the antagonist and *R*2 = radius of the pathogen.

### Specimens preparation for scanning electron microscope

The parasitism of hyphal cells of grapevine pathogen by *Trichoderma* was studied in detail by scanning electron microscopy (SEM). To obtain interaction sites of hyphae, PDA was inoculated at a constant distance from the edge of the Petri dish with a mycelial disc (5 mm) cut from the leading edge of a colony of *Trichoderma* and the pathogen. The mycoparasite and its host grew toward each other and their hyphae intermingled. After 48 h of incubation, the plate cultures were observed under a light microscope to verify the early stage of interaction. The interaction sites were marked and an agar block of 1 cm^2^ was removed for SEM preparation. Mycelial samples from the interaction region were fixed for 24 h with vapours of glutaraldehyde and osmium tetroxide (3:1), air dried for 48 h and sputter coated with gold.

### Data analysis

Specific activity of the cell-wall degrading enzymes and pathogenesis related enzymes (protease, chitinase and β-1,3-glucanase) were expressed as Unit/mg. The analysis of variance of enzymes activity and antagonistic activity were made using SPSS program var. 16.

## Results and discussion

### Phylogeny of the *Trichoderma* isolates based on analysis of multiple genetic loci

*Trichoderma* spp. are beneficial soil micro-organisms and potential candidates for biocontrol agents against soil-borne pathogenic fungi of grapevine plants. Extracellular enzymes such as chitinase, cellulase and β-glucanase produced by *Trichoderma* are important components of *Trichoderma* pathogens antagonism.

**Figure 1.**
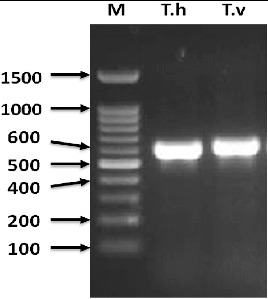
PCR products of 5.8S-ITS rDNA region from genus *Trichoderma harizianum* (lane 1) and *Trichoderma viride* (lane 2). Positions and sizes of 100 bp DNA ladder are shown on the left-hand side of the panel.

In this study, DNA sequencing of the 5.8S-ITS region of two parental isolates of protoplast fusion was carried out. The ITS region is one of the most reliable loci for the identification of a strain at a species level.[19,26] Therefore, 5.8S-ITS sequence analysis was used to establish the origin of *Trichoderma* isolates within the ascomycetes. An approximately 600 bp of 5.8S-ITS rDNA fragment was successfully amplified and sequenced from both parental *Trichoderma* isolates (Figure 1). Next, we performed a BLAST search with the *Trichoderma* spp. 5.8S-ITS rDNA gene to find most similar sequences in GenBank. The BLAST of the obtained sequence has identified these isolates as *T. harzianum* and *T. viride* and published in the GenBank with accession No. KF723609 and KF723610, respectively. By comparing the sequences of the 5.8S-ITS region to the sequences deposited in GenBank database, all of the *Trichoderma* isolates can be identified at species level with homology percentage of at least 99%. However, Mohammad [19] mentioned that GenBank database contain many sequences of *Trichoderma* isolates which may have been incorrectly identified. Hence, *Trich*OKEY search tool, a program that specifically compares ITS1 and ITS2 sequences to a specific database for *Trichoderma* generated from only vouchered sequences, was used to assess the reliability of BLAST results. *Trich*OKEY was recently used by many literatures and resulted in successful identification of *Trichoderma* isolates.[12,19] From the *Trich*OKEY results obtained, all isolates were identified, and the results were in agreement with the BLAST results.

**Figure 2.**
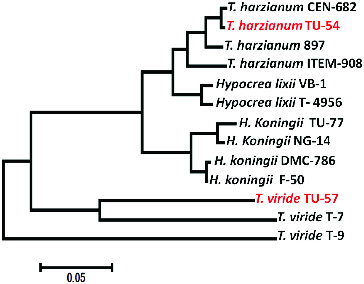
Phylogenetic tree and the diversity of 5.8S-ITS region sequences of two *Trichoderma* strains compared with some *Trichoderma* strains. Phylogenetic tree was generated using parsimony neighbour-joining and maximum likelihood analysis.

As can be seen from the phylogenetic tree given in Figure 2, the two parental isolates had almost 100% similarity with *Trichoderma harzianum* CEN-682 (accession No. KC576717.1) and 99% similarity with *Trichoderma viride* T-9 (accession No. HQ259986.1). The tree is based on the results of distance matrix analyses of all available 5.8S-ITS region primary structures for *Trichoderma* species. The topology of the tree was evaluated by performing maximum parsimony and maximum close-neighbour-interchange analyses of the full data-set and subsets, respectively. Only sequences that were at least 98% similar were used for treeing (Figure 2). The phylogenetic positions of *Trichoderma* isolates presented by partial sequences were roughly reconstructed by applying the parsimony criteria without changing the overall tree topology. Multifurcations indicate that a common branching order was not significantly supported by applying different treeing methods.

### Isolation and fusion of protoplasts

**Figure 3.**
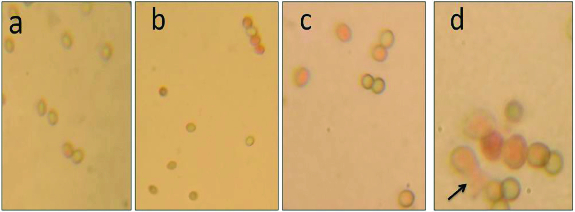
Protoplast fusion stages: (A) and (B) showing the released protoplasts from parent strains *T. harzianum* and *T. viride*, (C) and (D) showing fusion of protoplasts of parental strains after treatment with PEG.

Protoplast fusion is an effective tool for bringing genetic recombination and developing superior hybrid strains in filamentous fungi.[6,16,17] In this study, fungicides tolerance producing *T. harzianum* and *T. viride* were used for inter-specific protoplast fusion with the aim of enhancing the extracellular enzyme production as a method of biocontrol activity of *Trichoderma* spp. against some grapevine pathogens. Previously, El-Bondkly et al. [18] demonstrated the inter-specific crossing by protoplast fusion for genetic recombination in *Aspergillus niger* and *Trichoderma*. Furthermore,[12, 9] intra-specific hybridization in *T. harzianum* was achieved. In this study, the incubation of *Trichoderma harzianum* and *Trichoderma viride* mycelium with lysing enzymes L1412 resulted in lysis of the cell wall and release of protoplasts. The commercial lysing enzymes at 8 mg mL^−1^ prepared in STC (1.2 M sorbitol, 10 mM Tris-HCl pH 7.0, 10 mM CaCl_2_ buffer were used to release the protoplasts from *T. harzianum* and *T. viride* with 0.6 mol/L KCl as osmotic stabilizer. We have already optimized the conditions for releasing the protoplasts at our laboratory using different permutation combinations in various filamentous fungi including *Trichoderma*.[26] Swelling and rounding up of cell content were observed initially and subsequently. The *Trichoderma* mycelium started lysing after 2 h. Almost complete digestion of mycelia and release of protoplasts occurred prominently after 3 h of incubation (Figure 3(A) and 3(B)). Interestingly, the release of protoplasts was significantly affected by the concentrations of lysing enzymes. At low concentrations, the lysis of fungal mycelium took place only at the tip portion resulting in a minimum release of protoplasts, whereas at high enzyme concentrations, though the mycelium effectively lysed, the protoplasts bursted immediately after release and disintegrated. Among different concentrations of lysing enzymes tried, 8 mg mL^−1^ with 0.6 mol/L KCl as osmotic stabilizer was the optimized condition to release higher number of protoplast from different *Trichoderma* spp. However, Pe’er and Chet [21] obtained highest protoplasts from *T. harzianum* using Novozym 234 at 10 mg mL^−1^ with 0.6 mol/L KCl. Also, Fahmi et al. [6] used 15 mg mL^−1^ of Novozym with 0.6 mol/L sucrose to isolate maximum protoplasts from *T. harzianum* and *T. koningii*. Furthermore, Prabavathy et al. [7] obtained maximum number of protoplasts from *Trichothecium* spp. using Novozym 234 in combination with chitinase and cellulase each at 5 mg mL^−1^. Protoplasts fusion in *Trichoderma* has been achieved using 40% polyethylene glycol (PEG) that was already reported as optimum concentration for inter-specific fusion of protoplasts between *T. harzianum* and *T. longibrachiatum*.[15] On the other hand, Pe’er and Chet [21] used 33% PEG for intra-specific protoplast fusion in *T. harzianum*. When the protoplasts were mixed with PEG solution, they attracted each other and pairs of protoplasts were observed and seen under phase contrast microscope, which were subsequently fused together (data not shown). The concentration of PEG is highly critical for effective fusion of protoplasts. Higher concentrations of PEG caused shrinking and bursting of protoplasts.[7,16] The concentration between 40% and 60% was suitable for protoplasts fusion in different fungi.[7] Although aggregation of more than two protoplasts has occurred, fusion was observed between only two protoplasts. Later, the plasma membranes in the place of contact of fused protoplasts dissolved and fusion of protoplasmic contents took place (Figure 3(C) and (D)). Subsequently, the nuclei of the pairing protoplasts fused together (karyogamy) in many cases and in some cases, dikaryotic stage without nuclear fusion was observed. Finally, the fused protoplasts became single, larger in size and round- or oval-shaped structures.

### Regeneration of fused protoplasts and selection of fusants

**Figure 4.**
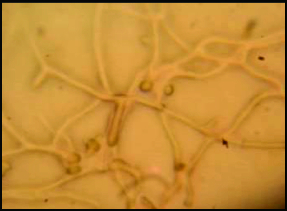
Regeneration of fused protoplasts between *T. harzianum* and *T. viride* after incubation for 36 h on PRMM medium supplemented with two fungicides.

The fused protoplasts started regenerating after two days (Figure 4) and developed mycelium after three days on selection media of PRMM medium supplemented with two selective fungicides for further selection. Though we observed an initial set back in growth of fusants, the colonies exhibited fast mycelial growth after three days. On the other hand, the parental protoplasts could not germinate on selective medium. Moreover, as previously reported,[16] the protoplasts that formed clumps were not viable and failed to germinate into colonies. At the first subculture, most of the inter fusants grew very fast on PDA as compared to the parents. These indicate the quicker adaptability of the fusion strains in the new environment. All the fusant strains grew luxuriantly and sporulated profusely compared to the parents. The intensity of yellow pigmentation in fusant strains was high as compared to parents. This data were in agreement with Prabavathy et al.[7] Colony development was observed after four days. Based on the mycelial growth, 21 strong growing colonies of fusants were selected and designated as F 1 to F 17. On the other hand, the non-fusion protoplasts could not germinate into colonies even after three days on selective medium. The regenerated protoplasts from the two parents were morphologically presented as dark green dense mycelium and thick dense green mycelium. Interestingly, the fusant colonies that appeared on third day after plating on selective medium were morphologically presented as white and green mycelium, very thick greenish yellow, sparse yellowish brown, white and green mycelium and thick dense green mycelium (Table 1). The pigmentation of parental strains was dark green and yellow but their fusants pigment were ranged from dark green to off-white. Also, the spores colouration of parental strains and their fusants were ranged from light green to green (Table 1).

**Table 1:** Colony morphology, pigmentation and sporulation of fusant and parent strains.

Strains	Morphology	Pigmentation	Spore colouration
P1 (*T. harzianum*)	Dark green dense mycelium	Dark green	Light green
P2 (*T. viride*)	Thick dense green mycelium	Yellow	Green
F 1	White and green mycelium	Yellow	Green
F 2	Thick green and white mycelium	Yellow	Green
F 3	Thick dense green mycelium	Green	Light green
F 4	Thick green and white mycelium	Dark green	Green
F 5	Thick dense green mycelium	Yellow	White green
F 6	Thick dense green mycelium	Green	Green
F 7	Very thick greenish yellow	Dark green	Light green
F 8	White and green mycelium	Green	Green
F 9	Sparse yellowish brown	Dark green	Green
F 10	Thick dense green mycelium	Yellow	White green
F 11	Thick green and white mycelium	Green	Green
F 12	Thick dense green mycelium	Green	Light green
F 13	Thick green and white mycelium	Dark green	Green
F 14	Very thick greenish yellow	Green	Green
F 15	White and green mycelium	Dark green	Green
F 16	Sparse yellowish brown	Off-white	White green
F 17	Very thick greenish yellow	Yellow	Green

### Enzymes activity of *Trichoderma* parents and fusants

The β-1,3-glucanase activity increased about threefold with fusant 15 (Fus. 15) (Table 2). It remarkably increased for all fusants except Fus. 1 and Fus. 2. Chitinase activity was estimated with 58% of the fusants and more than threefold increase in enzyme activity was recorded with four fusants, Fus. 3, Fus.13, Fus.15 and Fus.16, as compared to the parental isolates. Also, it was decreased with Fus. 6 and Fus. 7. Moreover, protease activity was moderately increased and two fusants record significant increases (Fus. 3 and Fus. 8). The maximum enzyme activity of 2.21 units was estimated in culture filtrate of the Fus. 3 and the minimum (1.29 unit) was recorded in Fus. 12. On the other hand, protein contents were recorded highly increased with Fus. 16 (322.3 μg ml^−1^) and it was significantly decreased with Fus. 3 and Fus. 8 (184.3 and 191.7 μg ml^−1^). In general, most of the *Trichoderma* fusants recorded an increase in enzyme activity comparing with *Trichoderma* parental isolates. In contrast with the parental strains, the enzymes activity such as β-1,3-glucanase, chitinase and protease were prominent and higher in most of the fusants than the parents indicating the improved production of these enzymes in those fusants (Table 2). The higher production of β-1,3-glucanase, chitinase and protease were confirmed by quantitative assays. There was a threefold increase in enzyme activity of some fusants. A variety of extracellular lytic enzymes may play an important role for the parasite. High chitinase and β-glucanase activities have been reported to be produced by *T. harzianum* [12] and there may be a relationship between the production of these enzymes and the ability to control plant diseases.[2,6,27] Although, majority of the fusants had shown enhanced enzyme activity, a few fusants exhibited decrease in activity as compared to the parents. This data had previously been observed as well.[18] These results indicate that partial or incomplete genetic recombination might be taking place during protoplast fusion, which could have led to negative effects in some fusants. These results were in agreement with those obtained.[7–9]

**Table 2:** Enzymes activity of *Trichoderma* parents and fusants isolates.

Strains	β-glucanase activity (U/ml)	Chitinase activity (U/ml)	Protease activity (U/ml)	Protein content (μg ml^−1^)
P1 (*T. harzianum*)	2.93^f^	0.39^f^	1.93^c^	223.7^g^
P2 (*T. viride*)	3.67^e^	0.45^e^	2.05^b^	229.3^f,g^
F 1	2.49^g^	0.36^f^	1.84^d^	204.0^i^
F 2	2.70^f^	0.37^f^	1.97b^c^	205.7^i^
F 3	3.57^e^	1.22^c^	2.21^b^	184.3^k^
F 4	4.40^d^	0.46^e^	1.93^c^	212.7^h^
F 5	4.83^c^	0.38^f^	1.47^g^	272.3^c^
F 6	3.74^e^	0.29^g^	1.57^f^	263.3^d^
F 7	1.91^h^	0.28^g^	1.83^d^	242.0^e^
F 8	3.28^e^	0.66^d^	2.20^b^	191.7^j^
F 9	4.57^c,d^	0.55^e^	1.54^f^	286.3^b^
F 10	3.45^e^	0.65^d^	1.85^d^	207.3^i^
F 11	2.26^g^	0.50^e^	2.00^b^	232.0^f^
F 12	3.02^f^	0.86^d^	1.69^e^	272.7^c^
F 13	5.59^b^	1.18^c^	2.28^a^	215.0^h^
F 14	3.25^e^	0.29^g^	1.45^g^	290.7^b^
F 15	6.32^a^	1.35^b^	2.35^a^	242.0^e^
F 16	2.09^g,h^	1.81^a^	1.62^e^	322.3^a^
F 17	2.86^f^	0.74^d^	1.29^h^	328.0^a^

Note: The same letters within a column indicate that the values are not significantly different at the *p* = 0.01 level.

Antagonistic effects of all *Trichoderma* parental and fusant isolates were tested against *Macrophomina phaseolina*, *Pythium ultimum* and *Sclerotium rolfsii* on PDA at 28 °C after seven days (Table 3). The antagonistic capacities were tested using dual culture method. In all the dual culture plates, the contact zone appeared as a curve, with concavity oriented towards pathogens. The curvature of the contact area between the colony of antagonistic fungi and the colony of pathogenic fungi in the same PDA plate depends on the growth rate of the colonies. If one colony has a faster growth rate than the other, a curve in the contact zone will most probably be observed. However, if the two colonies have the same growth rate, a straight line would be observed when mycelia from both fungi come into contact.[5,9,27] The averaged inhibition percentage (*I*%) of mycelial growth for grapevine pathogens were presented in Table 3. Among all *Trichoderma* fused isolates, Fus. 2 exhibited the lowest inhibition to the mycelial growth of *M. phaseolina* with an inhibition percentage of 57.1%, whereas Fus. 15 showed the highest inhibition percentage (92.8) against the growth of *M. phaseolina*. In the antagonistic test against *P. ultimum*, Fus. 1 exhibited the lowest antagonistic capacity with inhibition percentage of 57.1% while Fus. 15 exhibited the highest percentage of inhibition, 92.8%. Moreover, Fus. 1 exhibited the lowest inhibition of the mycelial growth of *S. rolfsii* with an inhibition percentage of 66.7% whereas Fus. 9 showed the highest inhibition percentage (98.2) against the growth of *S. rolfsii.* Overall, all *Trichoderma* isolates showed the ability to inhibit the mycelial growth of grapevine pathogens with at least 57.1%.

**Table 3.** Antagonistic activity of *Trichoderma* parents and fusion against some grapevine pathogens.

Strains	*Macrophomina phaseolina*	*Pythium ultimum*	*Sclerotium rolfsii*
P1 (*T. harzianum*)	82.8^b,c^	82.1^d^	91.2^c^
P2 (*T. viride*)	71.4^e^	60.7^h^	84.2^d,e^
F 1	82.1^c^	57.1^i^	66.7^h^
F 2	57.1^h^	78.6^e^	77.2^f^
F 3	82.1^c^	78.6^e^	80.7^e^
F 4	75.0^d^	71.4^g^	73.6^g^
F 5	75.0^d^	89.3^b^	80.7^e^
F 6	82.1^c^	75.0^f^	91.2^c^
F 7	78.6^c,d^	75.0^f^	77.2^f^
F 8	67.8^f^	82.1^d^	77.2^f^
F 9	85.7^b^	92.8^a^	98.2^a^
F 10	71.4^e^	85.7^c^	94.7^b^
F 11	75.0^d^	78.6^e^	84.2^d,e^
F 12	67.8^f^	82.1^d^	94.7^b^
F 13	85.7^b^	82.1^d^	76.8^f^
F 14	67.8^f^	71.4^g^	94.7^b^
F 15	92.8^a^	92.8^a^	80.7^e^
F 16	60.7^g^	75.0^f^	84.2^d,e^
F 17	67.8^f^	71.4^g^	87.8^d^

Note: The same letters within a column indicate that the values are not significantly different at the *p* = 0.01 level.

Interestingly, all *Trichoderma* isolates exhibited inhibition of the mycelial growth of all pathogens. This could be due to the production of diffusible components, such as lytic enzymes or water-soluble metabolites.[3,11,12,22] These components, such as chitinases and glucanases, were always secreted by *Trichoderma* in low levels. Therefore, they can act against the pathogenic fungi before mycelial contact, thus increasing the antagonism of *Trichoderma*. After *Fusarium* cell-wall degradation, the released components will induce the gene expression related to mycoparasiticism, allowing *Trichoderma* to be more antagonistic.[5,6,11,12]

### SEM observation of the mycoparasitic nature of *Trichoderma*

**Figure 5.**
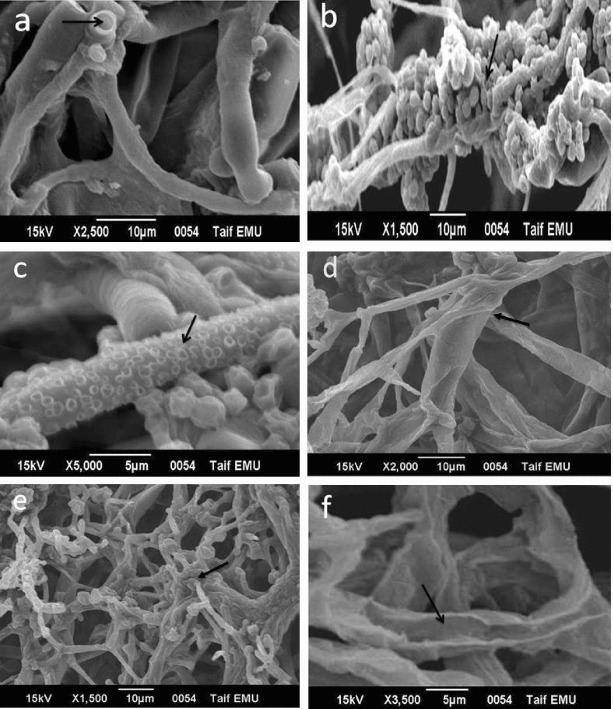
Scanning electron micrographs showing antagonistic activity of *Trichoderma* fusants. (A) and (B) Coiling of *Trichoderma* fusant (Fus. 15) and spores formation over *M. phaseolina.* (C) Fus. 15 formations of aspersorium-like structures without penetrating the cell wall of *S. rolfsii*. (D) and (E) Coiling of *Trichoderma* fusant (Fus. 15) and spores formation over lytic enzymes of *P. ultimum*. (F) Fus. 15 seem to be capable of degrading the cell walls of *P. ultimum*.

A more detailed picture of the development of coils and initiation of interaction structures between the two strains was obtained by SEM (Figure 5). Hypha coiling was observed in all *Trichoderma* strains but only Fus. 15 was chosen for visualization via SEM. Scanning electron micrographs after three days of the fusion showed complete colonization of *M. phaseolina* with *Trichoderma* strain. The contact zone revealed that the parasitic hyphae reached and grew on the surface always with coiling and spores formation over *M. phaseolina* (Figure 5(A) and 5(B)) and later they form aspersorium-like structures without penetrating the cell wall of *S. rolfsii* (Figure 5(C)). The *S. rolfsii* invaded hyphae looked disintegrated. A collapse in the parasitized hyphae was common with coiling and penetration of *Trichoderma* fusant (Fus. 15) and spore formation over lytic enzymes of *P. ultimum* (Figure 5(D) and 5(E)). Furthermore, total disappearance of the host hyphae after seven days of the interaction was observed. Lytic enzymes seem to be capable of degrading the cell walls of *P. ultimum* (Figure 5(F)) as is the strain (Fus. 15), which produces high amounts of β-glucanase.

## Conclusion

In conclusion, this study primarily elucidates that protoplast fusion of two fungicide tolerant mutants of *T. harzianum* and *T. viride* enhances β-glucanase, chitinase and protease enzyme activity in fusant compared to the parental strains, and there is an antagonistic activity development in most *Trichoderma* fusant isolates against grapevine pathogens *M. phaseolina*, *P. ultimum* and *S. rolfsii*.
